# Efficient oxidation of sulfides to sulfoxides catalyzed by heterogeneous Zr-containing polyoxometalate grafted on graphene oxide

**DOI:** 10.1038/s41598-023-43985-z

**Published:** 2023-10-05

**Authors:** Zahra Yekke-Ghasemi, Majid M. Heravi, Masoume Malmir, Masoud Mirzaei

**Affiliations:** 1 Department of Organic Chemistry, Faculty of Chemistry, Alzahra University, Tehran, Iran; 2 Department of Chemistry, Faculty of Science, Ferdowsi University of Mashhad, Mashhad, 9177948974 Iran; 3 Khorasan Science and Technology Park (KSTP), 12Th Km of Mashhad-Quchan Road, MashhadKhorasan Razavi, 9185173911 Iran

**Keywords:** Catalyst synthesis, Catalytic mechanisms, Heterogeneous catalysis

## Abstract

In this study, a tri-component composite named **Zr/SiW**_**12**_**/GO** was meticulously prepared through an ultrasonic-assisted method. This composite incorporates zirconium nanoparticles (Lewis acid), a negatively charged Keggin type polyoxometalate, and graphene oxide, and serves as a remarkable heterogeneous catalyst. The Keggin component plays multiple roles as reducing, encapsulating, and bridging agents, resulting in a cooperative effect among the three components that significantly enhances the catalytic activity. The catalytic performance of **Zr/SiW**_**12**_**/GO** was thoroughly investigated in the oxidation of sulfides to sulfoxides under mild conditions, employing H_2_O_2_ as the oxidant. Remarkably, this composite exhibited exceptional stability and could be effortlessly recovered and reused up to four times without any noticeable loss in its catalytic activity.

## Introduction

The incorporation of sulfoxide moiety is of great significance in the structure of biomolecules and synthetic intermediates, which are widely utilized in the production of various biological and chemical products^1,2^. The oxidation of sulfides to sulfoxides represents the simplest synthetic route for obtaining sulfoxides, and numerous oxidative reagents and methods have been developed for this conversion^3,4^. However, the pursuit of selective catalytic oxidation using environmentally friendly oxidants is a crucial and demanding objective for the chemical industry^5,6^, with dilute hydrogen peroxide being one such oxidant^7^. The favorable properties of aqueous hydrogen peroxide have prompted the development of several valuable methods for the oxidation of sulfides to sulfoxides and sulfones, employing diverse catalysts. Recently, a wide range of catalysts containing transition metals incorporated within frameworks or grafted onto solid surfaces have been prepared and explored for various oxidations in the presence of H_2_O_2_^8–16^. Among these catalysts, those containing metal species^17–20^, particularly zirconium, have demonstrated remarkable catalytic activity. Nevertheless, these systems have exhibited certain drawbacks, such as the requirement for the recovery of expensive catalysts, presence of residual metals in the final products, and formation of allylic oxidation products. Consequently, the development of a novel, environmentally friendly method that can overcome these challenges represents a highly challenging task.

Polyoxometalates (POMs) are a subset of metal-oxide clusters that exhibit a wide range of sizes, ranging from nanometers to several micrometers. Their diverse structures, sizes, photochemistry, redox chemistry, and charge distribution make them crucial in emerging fields such as sensors^21^, catalysts^22–24^, medicine^25^, magnetism^26^ and batteries^27^. The high stability of the redox states of POMs allows them to act as electron reservoirs, giving rise to mixed valence state species that are important in their use as catalysts^28^. Generally, POMs are formed from combinations of metal oxide building blocks with a general formula of [MOx]n (M is referred to as the addendum atom and is a transition metal), creating anionic clusters that can also contain cations (known as heteroatoms) such as B^3+^, Si^4+^ and P^5+^ further diversifying and complicating their chemistry. Based on the presence or absence of heteroatoms, POMs can be categorized as heteropolyanions or isopolyanions.

Keggin-type polyoxoanions with the formula [XM_12_O_40_]^n−^ (X represents the heteroatom, and M represents the addendum atom) benefit from a unique and highly symmetrical structure that exhibits exceptional stability under various conditions, making them a subject of extensive study. Furthermore, by altering the constituent metals, their thermal or chemical stability and acidity properties at the atomic/molecular level can be controlled without affecting the Keggin structure. Keggin-type polyoxoanions have been widely employed in several catalytic applications due to their significant characteristics. For example, their large negative charges enable them to act as Lewis bases, and when partially or fully protonated, they can facilitate acid-catalyzed reactions as Brönsted acids, such as esterification and hydrolysis. The presence of certain metal ions with unoccupied orbitals allows them to act as Lewis acids, and their ability to undergo reversible, multi-electron redox processes makes them suitable catalysts for the oxidation of alcohols, alkanes, and olefins^29^. It is important to note that Keggin-type POMs, like other members of their family, are generally soluble in aqueous solutions, making catalyst recovery challenging from these media. To overcome this issue, heterogeneous systems such as the formation of composites or hybrids of POMs using solid supports with high surface areas like carbon nanomaterials or MOFs have been actively explored^30–32^. The use of larger-sized cations or organic cations, such as dimethyldioctadecylammonium or tetrabutylammonium salts, for charge balance has also been investigated. Notably, Zr-containing POMs have exhibited pronounced catalytic activity in various reactions, including oxidation reactions, due to the presence of open coordination sites on the Zr center^13,33–35^.

Composites comprising of POMs, metal nanoparticles (NPs), and carbon nanomaterials can be formed through covalent linkage, non-covalent interactions, or electrostatic attachment methods, such as layer by layer assembly. For instance, POMs can be incorporated into graphene (G) or graphene oxide (GO) networks using a green and straightforward wet-chemical synthesis approach, wherein the POMs undergo a redox reaction, transitioning from the reduced polyblue form to a neutral form, and acting as intermolecular linkers.

**Figure 1:**
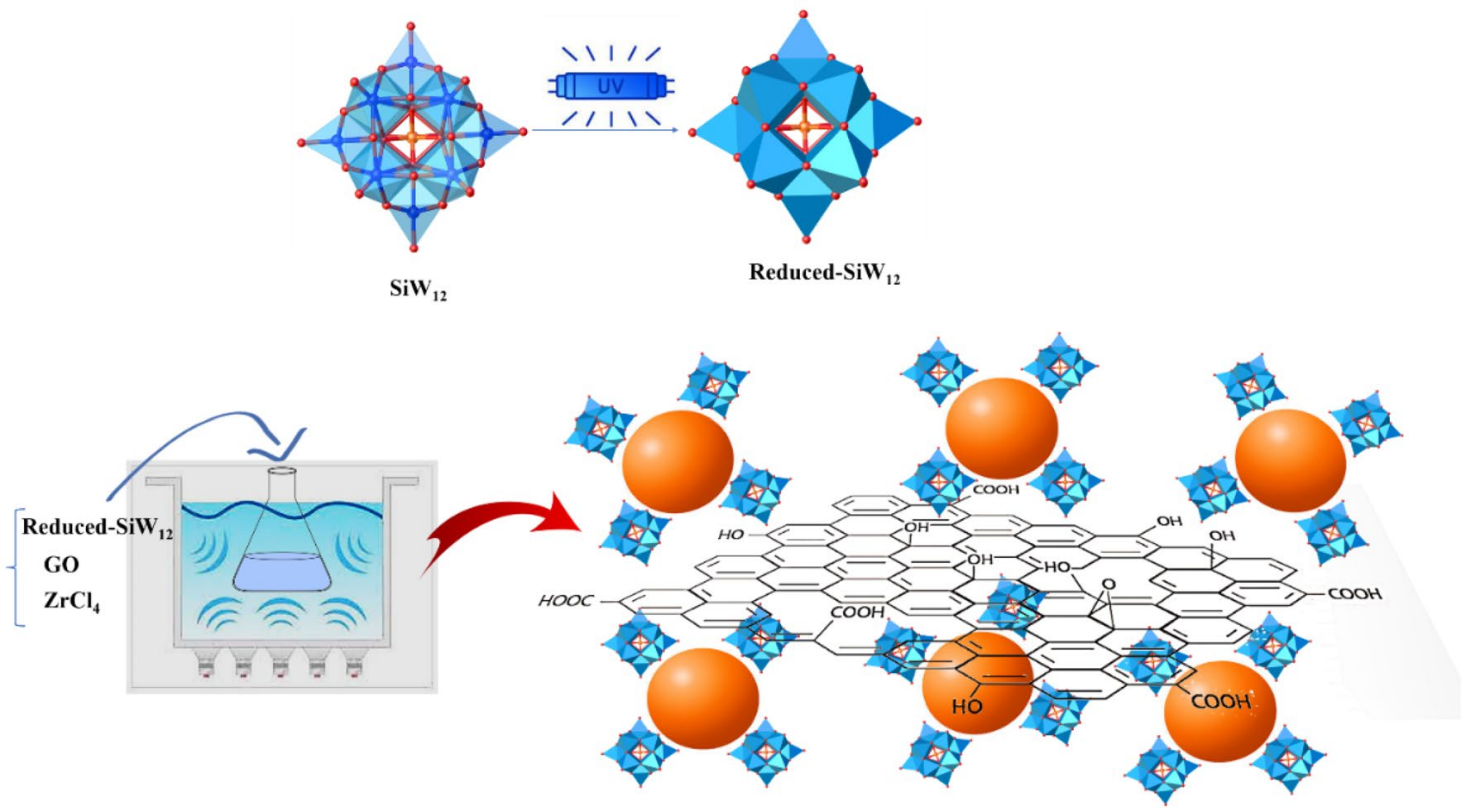
Synthetic route of **Zr/SiW**_**12**_**/GO** tricomponent catalyst with representation of SiW_12_ (polyhedral representation), Zr (orange ball representation), and GO.

In our ongoing investigation of the preparation and application of heterogeneous catalysts^36–43^, with a particular focus on POM-based catalysts^44–46^, we present a tri-component composite heterogeneous **Zr/SiW**_**12**_**/GO** catalyst for the oxidation of sulfides to sulfoxides using hydrogen peroxide. As anticipated, **Zr/SiW**_**12**_**/GO** functions simultaneously as a Lewis acid/base, enhancing catalytic activity by combining the zirconium Lewis acid with the negatively charged Keggin species on the GO support (Fig. 1). Additionally, we have examined the recyclability of the **Zr/SiW**_**12**_**/GO** catalyst to assess its nature and stability.

## Result and discussion

### Synthesis and characterization of catalysts

The tricomponent **Zr/SiW**_**12**_**/GO** composite was synthesized by a simple reaction in an ultrasonic bath (Fig. 1). During synthesis, the introduction of the GO support into the composite can effectively increase the specific surface area of the composite for the dispersion of Zr nanoparticles and SiW_12_ species with the electrostatic interaction between negatively charged, reduced SiW_12_ units preventing the Zr NPs from aggregating. This agrees with prior reports that smaller Zr NPs rather than POM units can be affixed to the walls of the GO by the selection of the proper concentration of the reduced SiW_12_^47,48^.

**Figure 2:**
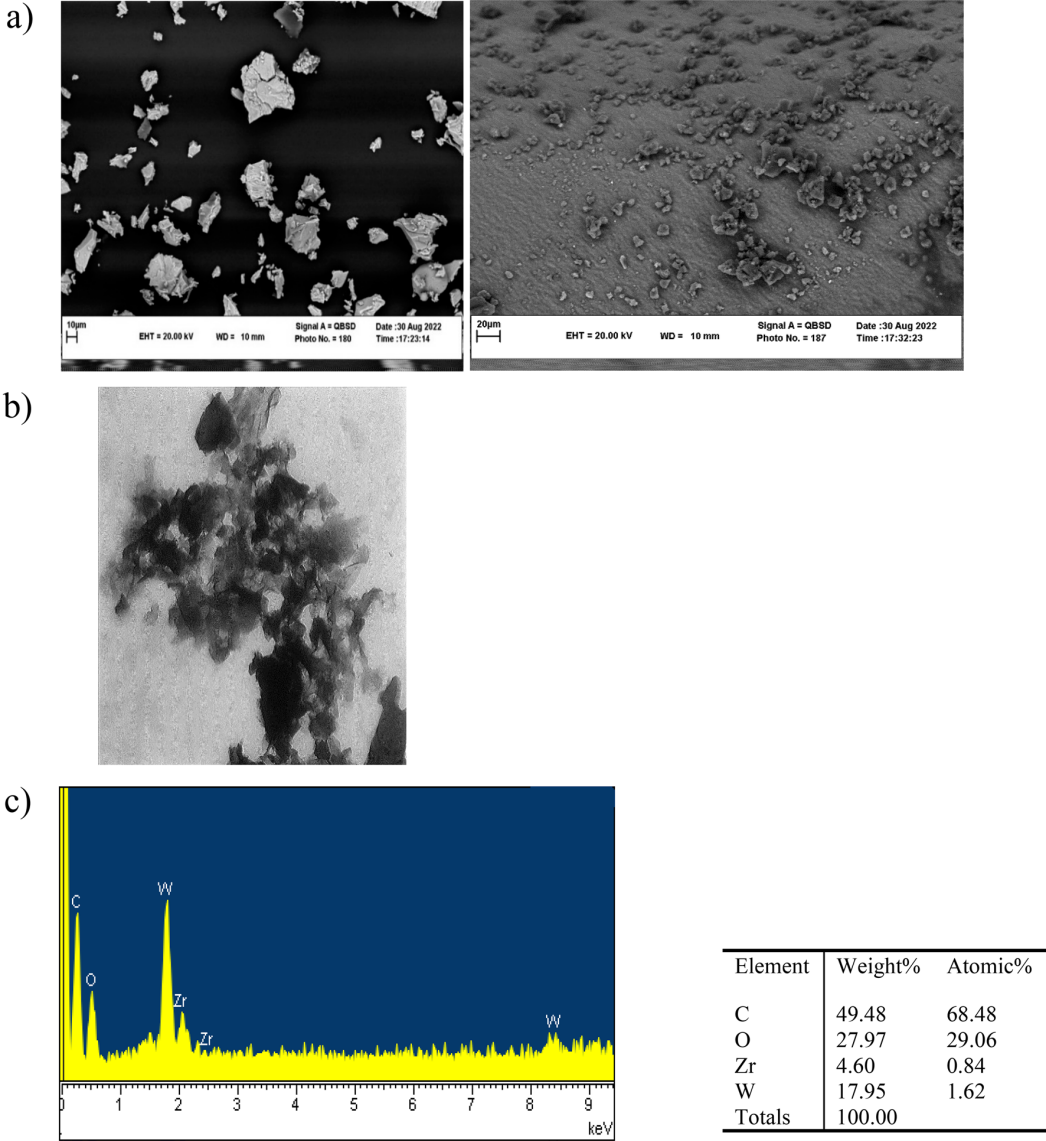
(**a**) SEM images of GO (right) and **Zr/SiW**_**12**_**/GO** composite (left); (**b**) TEM image of **Zr/SiW**_**12**_**/GO** composite; (**c**) EDX analysis of **Zr/SiW**_**12**_**/GO**.

Scanning electron microscopy (SEM) and transmission electron microscopy (TEM) were employed to ascertain the structure and morphology of the GO and Zr/SiW_12_/GO composite (Fig. 2a).

In situ energy dispersive X-ray analysis (Fig. 2c) showed strong tungsten and carbon peaks accompanied by Zr peaks, confirming the existence of all three components in this composite. The TEM observations are consistent with these results (Fig. 2b).

**Figure 3:**
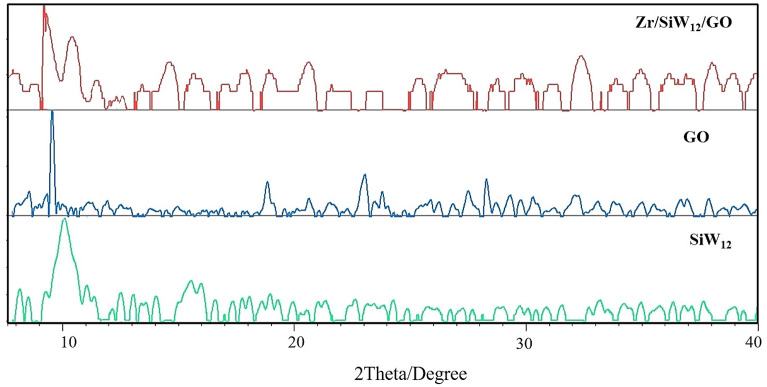
XRD patterns of the **Zr/SiW**_**12**_**/GO** composite, GO and SiW_12_.

The powder XRD patterns of SiW_12_, GO, and the **Zr/SiW**_**12**_**/GO** composite are depicted in Fig. 3. The GO exhibits a distinct diffraction peak at 9.8°, corresponding to the interlayer spacing of C (002) at 0.790 nm. The absence of any peak at a 2*θ* value of 26.5° confirms the complete oxidation of G. Moreover, the **Zr/SiW**_**12**_**/GO** composite displays diffraction peaks for both SiW_12_ and GO (notably, the characteristic peaks at 9°–11° for 2*θ* value), providing evidence for the formation of a tricomponent composite.

**Figure 4:**
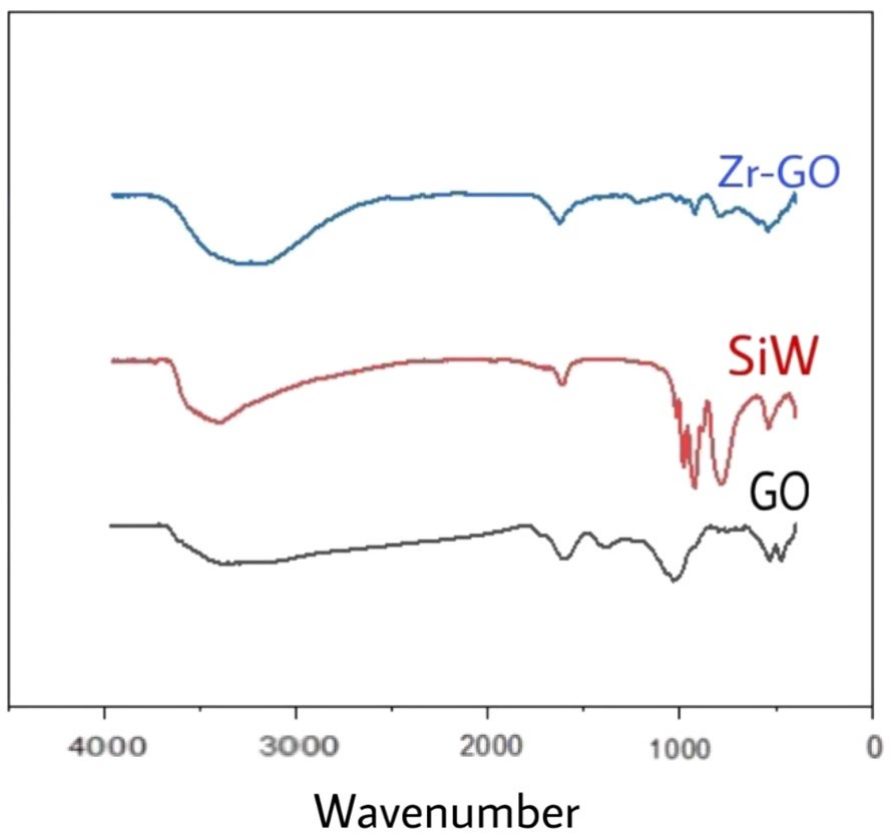
FTIR spectra of SiW_12_, GO and the **Zr/SiW**_**12**_**/GO** composite.

Furthermore, FT-IR spectra were obtained to analyze the chemical structure of the **Zr/SiW**_**12**_**/GO** composite. These spectra exhibit a vibration pattern akin to that of SiW_12_ and GO individually, thereby confirming the presence of both constituents within the composite (as shown in Fig. 4). The characteristic bands in the range of 600 − 1100 cm^−1^ can be attributed to the vibrations of SiW_12_, with strong bands observed for W–O_c_, W–O_t_, W–O_b_ and Si–O stretching vibrations at approximately 875, 801, 970, and 921 cm^−1^, respectively. Additionally, the bands at 1221, 1436, 1520, and 1647 cm^−1^ correspond to the stretching vibration of C=C and C=O bonds in GO. Notably, the band associated with carboxylic acid groups in GO, originally observed at 1710 cm^−1^, is shifted to 1627 cm^−1^ in the composite. This shift indicates the formation of robust hydrogen bonds between the oxygen atoms of SiW_12_ and the hydroxyl groups on the carboxylic acids.

### Catalytic activity

The catalytic activity of the **Zr/SiW**_**12**_**/GO** composite was investigated for the oxidation of methylphenyl sulfide (MPS) to methylphenyl sulfoxide using aqueous hydrogen peroxide (30%) under solvent-free conditions at room temperature. In the absence of a catalyst, the reaction yielded only 45% of the desired product after 12 h (Table S1, entry 1). To enhance the reaction efficiency, MPS (1 mmol) was oxidized using **Zr/SiW**_**12**_**/GO** (10 mg) and H_2_O_2_ (2 eq.) at room temperature, resulting in the formation of 95% sulfoxide in a significantly shorter reaction time (Table S1, entry 3).

Further investigations were conducted to determine the active site responsible for the oxidation of MPS in **Zr/SiW**_**12**_**/GO**. Various catalysts including Zr-free POM^44,45^ and Zr salts were examined. The results indicate that the presence of Zr is necessary for the oxidation of MPS, as confirmed by running the reaction in the presence of bare GO. Notably, Zr has been reported as one of the most effective metals for promoting the oxidation of sulfides^9^. While Zr salts exhibit reasonable catalytic activity, their separation and recovery from the reaction mixture pose challenges. In contrast, **Zr/SiW**_**12**_**/GO** can be easily separated by filtration due to the presence of the GO support. Moreover, it demonstrated excellent reusability for up to four runs with consistently high yields. These findings suggest that the use of a substrate for heterogenization not only facilitates the separation process but also enhances the performance of the Zr active site, potentially attributed to the synergistic effect between GO, POM, and Zr.

To optimize the reaction conditions, various catalyst and oxidant loadings were investigated for the oxidation of MPS under S.F. and r.t. conditions (Table S1). Among the catalyst loadings tested (Table S1, entry 2–4), the use of 10 mg of **Zr/SiW**_**12**_**/GO** yielded the highest product yield (Table S1, entry 3), while higher and lower catalyst loadings resulted in lower yields. It is worth noting that sulfone is one of the main products in this reaction, as the oxidation of sulfides can be further converted into sulfone under continued oxidation conditions. However, when four equivalents of H_2_O_2_ were used, both products were obtained in impure form with different ratios, indicating that excessive oxidant leads to the production of side products and the progression of the oxidation process. Therefore, based on the results in Table S1, it was determined that 2.0 equivalents of H_2_O_2_ is the optimal amount. Additionally, MPS was oxidized with H_2_O_2_ using **Zr/SiW**_**12**_**/GO** in various solvents (2.5 mL), and it was observed that not using any additional solvent resulted in better yields.

The effectiveness of the **Zr/SiW**_**12**_**/GO** catalyst was further demonstrated by investigating the oxidation of different sulfides under the optimized conditions, revealing its efficacy for both aromatic and aliphatic sulfides (Table 1).

**Table 1:** Synthesis of sulfoxides by oxidation of sulfides over **Zr/SiW**_**12**_**/GO** using H_2_O_2_ under S.F. and r.t. conditions.


Entry	R^1^	R^2^	Time (min)	Yield (%)^a^
**1**	C_6_H_5_	Me	20	95
**2**	C_6_H_5_	Et	35	94
**3**	C_6_H_5_	C_6_H_5_	60	83
**4**	*p*-MeC_6_H_4_	Me	63	80
**5**	*p*-BrC_6_H_4_	Me	50	90
**6**	*p*-O_2_NC_6_H_4_	Me	40	87
**7**	*p*-HOC_6_H_4_	Me	30	90
**8**	Me	Me	60	90
**9**	Me	Et	70	77
**10**	CH_3_	H	65	82

Reaction condition: a mixture of sulfide (1 mmol), H_2_O_2_ (2 eq.) with 4 drops EtOH and Zr/SiW_12_/GO (10 mg) was stirred without any solvent.

^a^Isolated yields.

**Figure 5:**
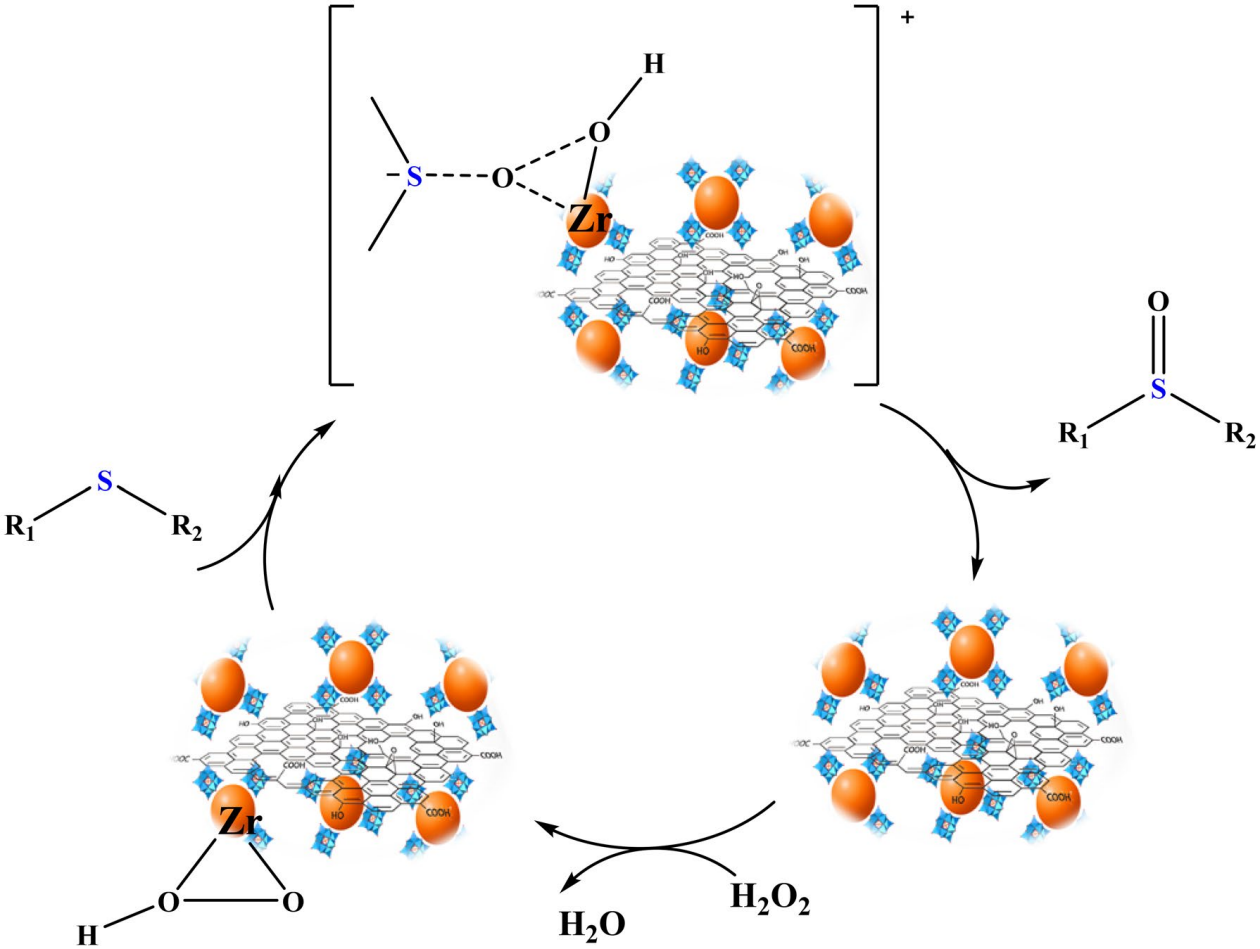
Schematic possible mechanism for the oxidation of sulfide catalyzed by **Zr/SiW**_**12**_**/GO** and H_2_O_2._

To gain insight into the mechanism, MPS oxidation over **Zr/SiW**_**12**_**/GO** and H_2_O_2_ without any additional solvents was examined. Similar mechanisms have been reported in recent literature for this reaction^12,49,50^. Figure 5 presents a possible mechanism for the catalytic oxidation of sulfide over the **Zr/SiW**_**12**_**/GO** composite. Initially, H_2_O_2_ can bind to **Zr/SiW**_**12**_**/GO**, forming an active electrophilic peroxide intermediate. Subsequently, this peroxide- **Zr/SiW**_**12**_**/GO** intermediate can undergo a nucleophilic attack by the -S atom of the sulfide, leading to the reduction of **Zr/SiW**_**12**_**/GO** back to its original state for subsequent runs, with the release of one mole of water. Notably, the presence of Zr is crucial in facilitating the formation of the peroxide-metal complex and activating H_2_O_2_ molecules^51–53^.

In the present study, the catalytic performance of the **Zr/SiW**_**12**_**/GO** composite was compared with other recent reports on the oxidation of sulfides to sulfoxides (Table 2, entries 1–6). The results demonstrated that the **Zr/SiW**_**12**_**/GO** composite exhibited superior performance in terms of reaction conditions and reaction times. Notably, this system offered the advantage of easy separation and high recyclability. The unique structure of SiW_12_, which was well-stabilized on the surface of GO, contributed to the formation of a heterogeneous structure, as confirmed by the hot filtration test results.

**Table 2:** Comparison of catalytic activity of **Zr/SiW**_**12**_**/GO** with other reported catalysts for the oxidation of MPS.

Entry	Catalyst (amount)	Conditions			Time (min)	Conversion (%)	Refs
		Oxidant	Solvent	Temp. or UV			
1	Mo_6_W_6_@EDMG (7 mg)	H_2_O_2_ (1.5 mmol)	EtOH	400 W lamp	120	88	^57^
2	MNP@TA‐IL/W (0.4 mol%)	H_2_O_2_ (1.5 e.q)	H_2_O	rt	60	98	^58^
3	Fe_3_O_4_@S‐ABENZ@VO (5 mg)	H_2_O_2_ (16 mmol)	S.F	rt	80	98	^59^
4	VO(BINE)@Fe_3_O_4_ (30 mg)	H_2_O_2_ (2 mmol)	S.F	rt	5	96	^14^
5	PAMAM-G1-PMo (50 mg)	H_2_O_2_ (0.55 mmol)	MeOH	rt	240	88	^60^
6	Zr/SiW_12_/GO (10 mg)	H_2_O_2_ (2 mmol)	S.F	rt	30	98	This work

### Reusability of the catalyst

I agreement with the recent report^54^, the recyclability of the **Zr/SiW**_**12**_**/GO** catalyst was investigated using the model reaction under optimal conditions. After completion of the reaction, the mixture was triturated with Et_2_O until it became clear, and the **Zr/SiW**_**12**_**/GO** catalyst was separated by filtration. Subsequently, the catalyst was dried in air and examined again in the model reaction. As shown in Fig. S1, the **Zr/SiW**_**12**_**/GO** catalyst could be successfully reused up to four times without significant loss of activity. However, when attempting to reuse the catalyst for the fifth reaction run, a dramatic decrease in catalytic performance was observed, resulting in a yield of only 65% for the model product. This decrease in activity can be attributed to the deposition of organic species on the catalyst during multiple reaction runs or the potential leaching of POM induced by NaBH_4_, which may disrupt the catalyst structure and subsequently reduce its catalytic activity. To investigate this observation further, the leaching of Zr was examined after four and five reaction runs. It was found that upon reusing the catalyst for four reaction runs, there was a remarkable increase in Zr leaching, providing an explanation for the observed lower catalytic activity.

**Figure 6:**
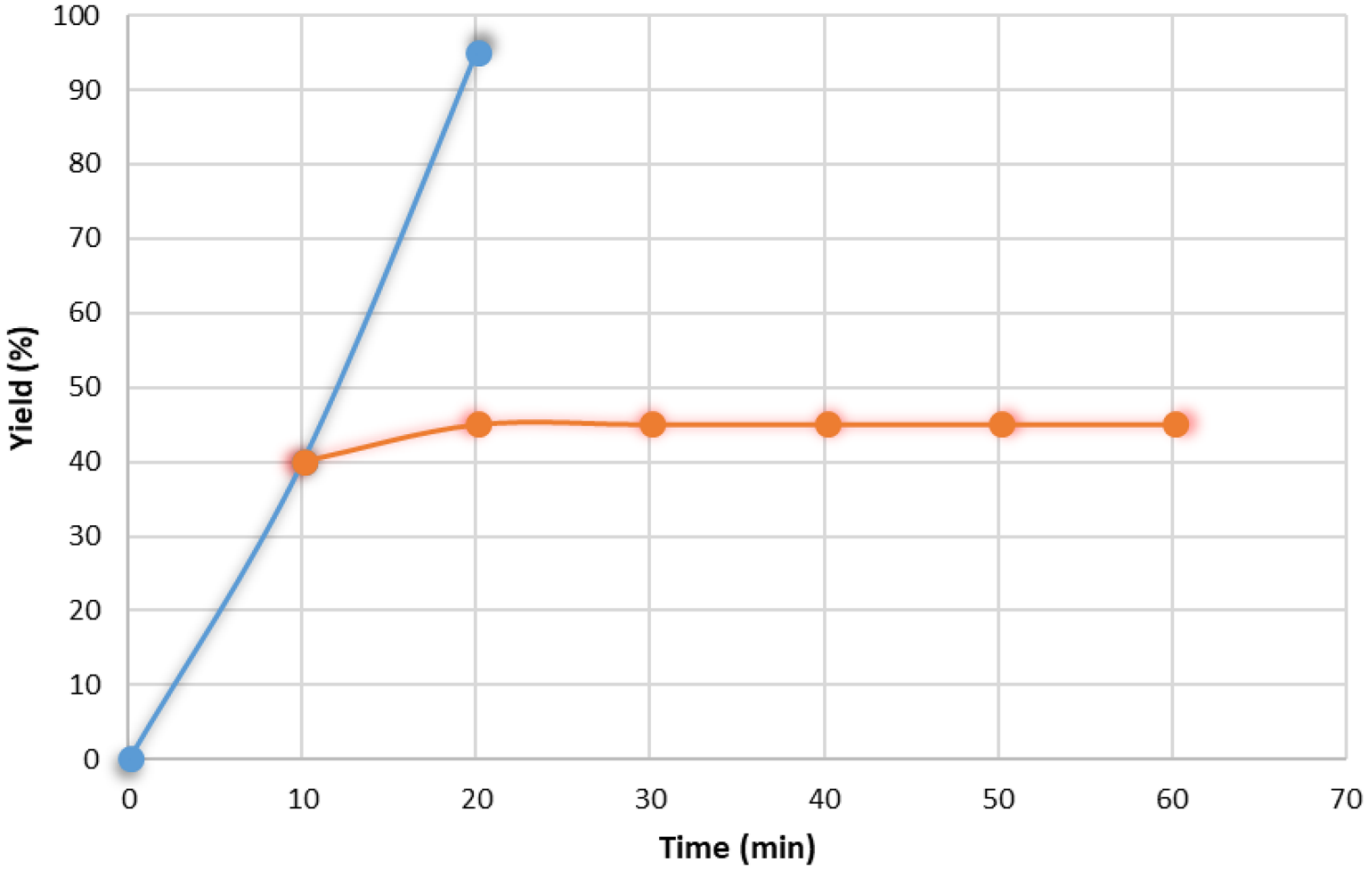
Monitoring of hot filtration experiment for the synthesis of MPS (blue line: with catalyst and red line: without catalyst).

Subsequently, a standard hot filtration experiment^55^ was conducted to ascertain the catalyst's composition for the synthesis of MPS under the optimal reaction conditions. The **Zr/SiW**_**12**_**/GO** catalyst was extracted after achieving 40% conversion (t = 10 min), allowing the reactants to continue reacting. The results revealed that, subsequent to the catalyst's removal, there was minimal leaching of Zr, and the conversion rate reached 45% even after 40 min. Furthermore, analysis using ICP-OES demonstrated a Zr content of 0.002 mmol g^−1^ in the filtrate, which is negligible when compared to the initial loading of 0.27 mmol g^−1^. These findings provided additional confirmation of the heterogeneous nature of the **Zr/SiW**_**12**_**/GO** catalyst (Fig. 6).

## Materials and methods

### Chemicals

All chemicals were purchased from Merck (www.merckmillipore.com) and Sigma-Aldrich (www.sigmaaldrich.com) and used as received. Oxidation were conducted using sulfides, H_2_O_2_, acetonitrile, methanol, ethanol, and deionized water.

### Instrumentation

The infrared spectra of the catalysts were recorded on a Thermo Nicolet/AVATAR 370 Fourier transform spectrophotometer. The morphology of the catalyst was studied by scanning electron microscopy (SEM) using a Leo 1450 VP, Germany instrument. Powder XRD patterns were obtained using a PANalytical B.V. diffractometer with Cu Kα radiation (λ = 1.54184 Å) at room temperature with a scan range 2θ = 5° to 50°, a step size of 0.05 °C and a step time of 1 s. The energy-dispersive X-ray (EDX) with resolution of about 500 nm (at an acceleration voltage of 10.00 kV and) was performed on a LEO-1450 VP unit (Zeiss, Germany).

### Preparation of catalyst

Graphene oxide (GO) was synthesized according to Kigozi, M. (2020) method and characterized by Powder XRD, SE M and FT-IR,^56^. The tricomponent catalyst was prepared with GO, reduced H_4_SiW_12_O_40_ (SiW_12_), and ZrCl_4_.

### Synthesis of Zr/SiW_12_/GO composite

An aqueous solution of SiW_12_ (0.5 mmol, 10 mL) was adjusted to the pH 1.18 and 1 mL of isopropanol was added. The solution was reduced photochemically with a UV light source (500 W Hg lamp) until the color of the solution become blue–black (approximate time = 30 min). The solution of reduced SiW_12_ added to GO (10 mL, and 8 mg mL^−1^) and ZrCl_4_ (0.5 mM) at room temperature in an ultrasonic bath. After 120 min of reaction, the tricomponent composites were assembled. The samples were centrifuged and then washed with H_2_O.

Yield: 0.26 g (13% based on W). FT-IR (KBr pellet, cm^−1^): 3272, 3184, 1627, 1510, 1231, 1081, 971, 924, 794, 746, 591, 544.

### Typical method for the catalytic oxidation of sulfides to sulfoxides

A 50 mL round bottom flask was charged with 1 mmol of sulfide, 10 mg of the **Zr/SiW**_**12**_**/GO** catalyst, and four drops of 96% EtOH. The resulting solution was stirred at room temperature, and 0.227 g (2 eq.) of 30 wt% H_2_O_2_ was slowly added. The reaction progress was monitored by TLC using a mixture of n-hexane and EtOAc (8:2) as the solvent system. Once the oxidation was complete, the **Zr/SiW**_**12**_**/GO** catalyst was filtered off and separated from the mixture by filtration. It was then washed with 95% EtOH and dried in a vacuum at room temperature overnight for recycling purposes. The reaction filtrate was washed with diethyl ether (3 × 7 mL), and the combined organic layers were dehydrated using Na_2_SO_4_. The crude products were purified by column chromatography on a silica gel column using n-hexane/ethyl acetate (8:2) as the eluent, resulting in the desired products. The ^1^HNMR spectrum for the corresponding methyl phenyl sulfoxide can be found online in the Supplementary Information (Fig. S2).

## Conclusion

In conclusion, we have successfully developed a sonochemical method for preparing a tri-component composite catalyst, **Zr/SiW**_**12**_**/GO**, for the catalytic oxidation of sulfides. This catalyst offers several advantages over previous solid supports, including high activity, stability, and improved recoverability. The incorporation of GO in the Zr-containing POMs provides enhanced recycling capability by stabilizing the active site dispersion. The combination of GO and Zr-containing POMs allows for effective oxidation and easy separation of the supported-catalyst active sites in the reaction system. The **Zr/SiW**_**12**_**/GO** catalyst can be easily recycled through filtration and reused up to four times without significant loss of activity.

### Supplementary Information

Supplementary Information.

## Data Availability

All data generated or analysed during this study are included in this published article [and its supplementary information files].
