# Supplementary material for: Efficient oxidation of sulfides to sulfoxides catalyzed by heterogeneous Zr-containing polyoxometalate grafted on graphene oxide
Source: Sci Rep. 2023 Oct 5;13:16752. doi: 10.1038/s41598-023-43985-z (PMC10556038; doi:10.1038/s41598-023-43985-z)
Supplement: Supplementary file 1 — Supplementary Information. [file 41598_2023_43985_MOESM1_ESM.pdf]

Supporting information for the manuscript

Table S1. Optimization of the reaction parameters and comparison of the catalytic performance of **Zr/SiW$_{12}$/GO** with Zr-free POM and Zr salts for oxidation of MPS[a]

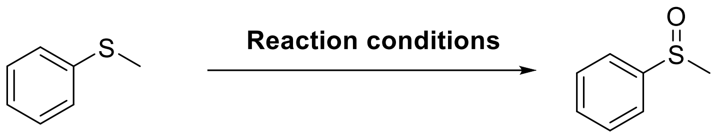

Entry	Catalyst (mg)	Conditions Oxidant (eq.)/ Solvent/ Temperature	Time (min)	Yield[b] (%)
1	Zr/SiW$_{12}$/GO (None)	H_2O_2 (2)/ S.F./ r.t.	720	45
2	Zr/SiW$_{12}$/GO (5)	H_2O_2 (2)/ S.F./ r.t.	20	80
3	Zr/SiW$_{12}$/GO (10)	H_2O_2 (2)/ S.F./ r.t.	20	95
4	Zr/SiW$_{12}$/GO (15)	H_2O_2 (2)/ S.F./ r.t.	20	95
5	Zr/SiW$_{12}$/GO (10)	H_2O_2 (1)/ S.F./ r.t.	20	70
6	Zr/SiW$_{12}$/GO (10)	H_2O_2 (1.5)/ S.F./ r.t.	20	80
7	Zr/SiW$_{12}$/GO (10)	H_2O_2 (None)/ S.F./ r.t.	20	60
8	Zr/SiW$_{12}$/GO (10)	H_2O_2 (4)/ S.F./ r.t.	20	Sulfoxide 35/ Sulfone 65
9	Zr/SiW$_{12}$/GO (10)	Pure O_2 / S.F./ r.t.	20	65
10	$ZrCl_4$ (10)	H_2O_2 (2)/ S.F./ r.t.	20	70
11	$Zr(NO_3)_4.5H_2O$ (10)	H_2O_2 (2)/ S.F./ r.t.	20	70
12	$Zr(SO_4)_2.4H_2O$ (10)	H_2O_2 (2)/ S.F./ r.t.	20	65
13	$Zr(CH_3COO)_4$ (10)	H_2O_2 (2)/ S.F./ r.t.	20	60
14	$(TBA)_7[PW_{11}O_{39}]$ (10)	H_2O_2 (2)/ S.F./ r.t.	20	40
15	$(TBA)_8[SiW_{11}O_{39}]\cdot 4H_2O$ (10)	H_2O_2 (2)/ S.F./ r.t.	20	30
16	$(TBA)_9[BW_{11}O_{39}]\cdot 11H_2O$ (10)	H_2O_2 (2)/ S.F./ r.t.	20	30
17	GO (10)	H_2O_2 (2)/ S.F./ r.t.	20	25
18	$ZrCl_4$/GO (10)	H_2O_2 (2)/ S.F./ r.t.	20	85
19	Zr/SiW$_{12}$/GO (10)	H_2O_2 (2)/ EtOH/ r.t.	20	70
20	Zr/SiW$_{12}$/GO (10)	H_2O_2 (2)/ H_2O/ r.t.	20	80
21	Zr/SiW$_{12}$/GO (10)	H_2O_2 (2)/ CH_3Cl/ r.t.	20	35

[a] MPS (1 mmol).

[b] Isolated yield.

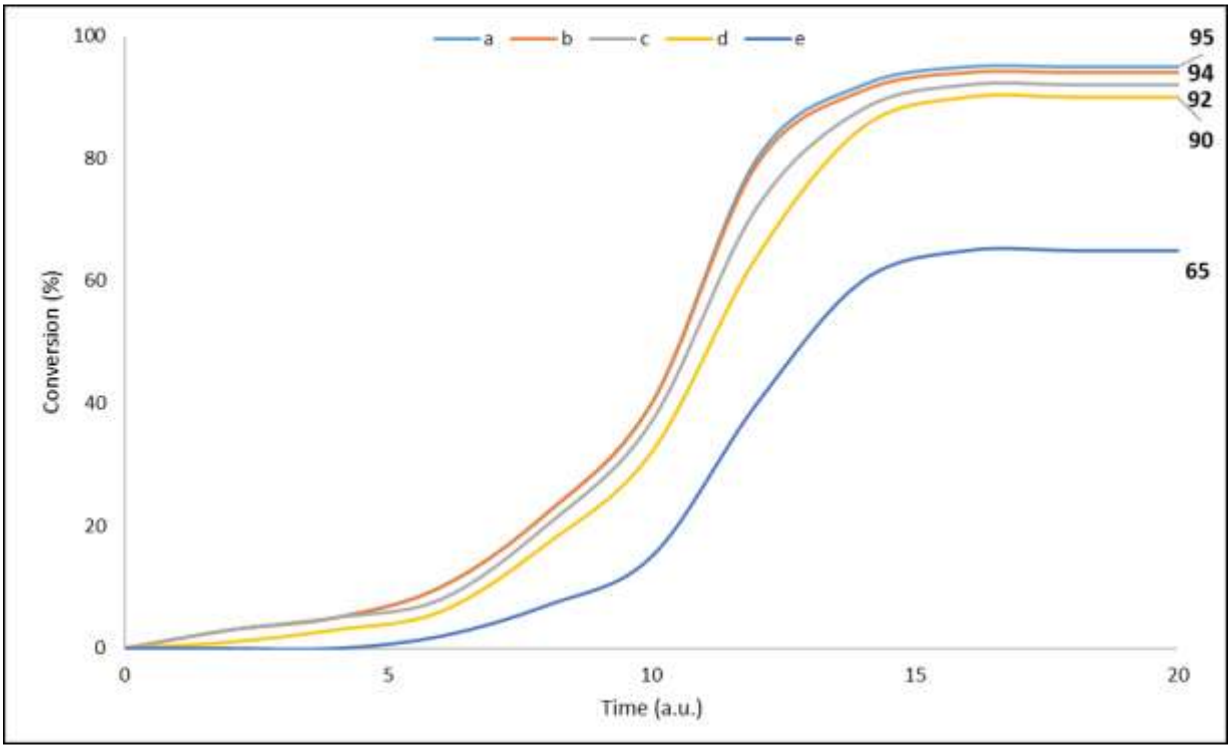

Figure S1. Investigating the performance of the recovered **Zr/SiW$_{12}$/GO** catalyst during the first to fifth cycles (a-e) in the oxidation of MPS by H_2O_2.

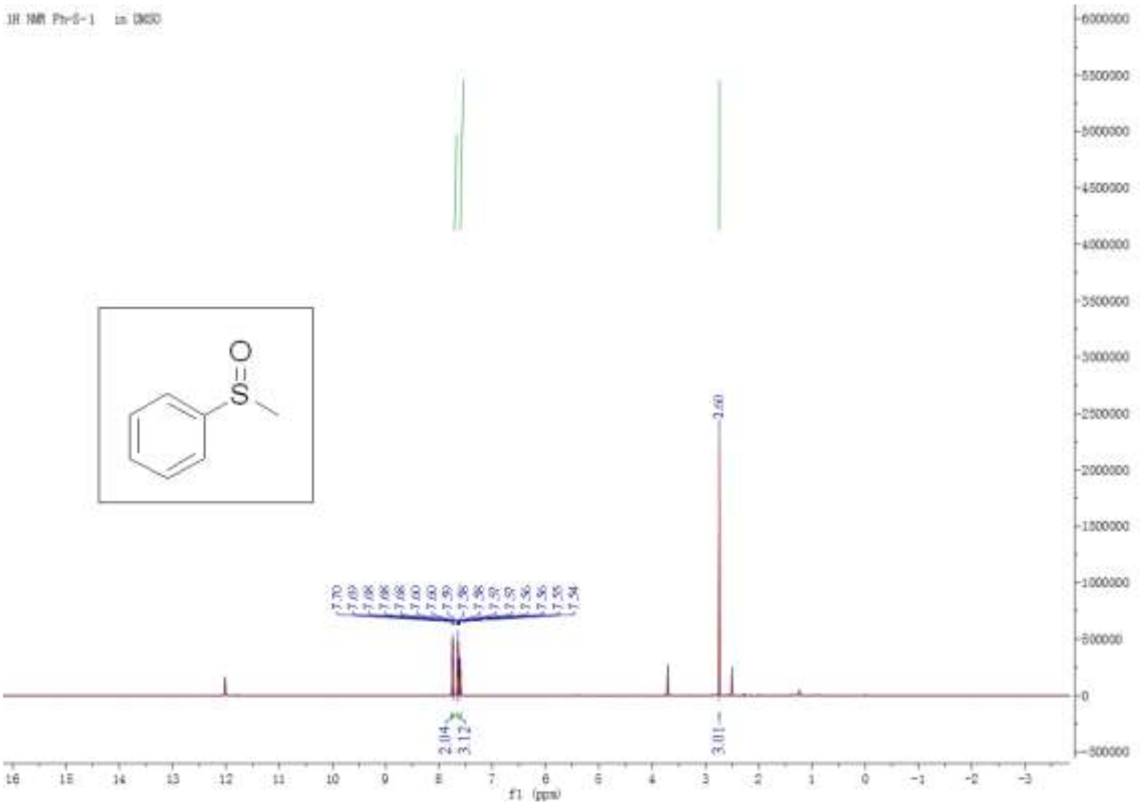

Figure S2. ^{1}HNMR spectra of MPS
